# The use of time-resolved fluorescence imaging in the study of protein kinase C localisation in cells

**DOI:** 10.1186/1471-2121-6-22

**Published:** 2005-04-26

**Authors:** Christopher D Stubbs, Stanley W Botchway, Simon J Slater, Anthony W Parker

**Affiliations:** 1 Department of Pathology and Cell Biology, Thomas Jefferson University, Philadelphia PA 19107 USA; 2 The Central Laser Facility, Rutherford Appleton Laboratory, CCLRC, Chilton, OX11 OQX UK

## Abstract

**Background:**

Two-photon-excitation fluorescence lifetime imaging (2P-FLIM) was used to investigate the association of protein kinase C alpha (PKCα) with caveolin in CHO cells. PKCα is found widely in the cytoplasm and nucleus in most cells. Upon activation, as a result of increased intracellular Ca^2+ ^and production of DAG, through G-protein coupled-phospholipase C signalling, PKC translocates to a variety of regions in the cell where it phosphorylates and interacts with many signalling pathways. Due to its wide distribution, discerning a particular interaction from others within the cell is extremely difficult

**Results:**

Fluorescence energy transfer (FRET), between GFP-PKCα and DsRed-caveolin, was used to investigate the interaction between caveolin and PKC, an aspect of signalling that is poorly understood. Using 2P-FLIM measurements, the lifetime of GFP was found to decrease (quench) in certain regions of the cell from ~2.2 ns to ~1.5 ns when the GFP and DsRed were sufficiently close for FRET to occur. This only occurred when intracellular Ca^2+ ^increased or in the presence of phorbol ester, and was an indication of PKC and caveolin co-localisation under these conditions. In the case of phorbol ester stimulated PKC translocation, as commonly used to model PKC activation, three PKC areas could be delineated. These included PKCα that was not associated with caveolin in the nucleus and cytoplasm, PKCα associated with caveolin in the cytoplasm/perinuclear regions and probably in endosomes, and PKC in the peripheral regions of the cell, possibly indirectly interacting with caveolin.

**Conclusion:**

Based on the extent of lifetime quenching observed, the results are consistent with a direct interaction between PKCα and caveolin in the endosomes, and possibly an indirect interaction in the peripheral regions of the cell. The results show that 2P-FLIM-FRET imaging offers an approach that can provide information not only confirming the occurrence of specific protein-protein interactions but where they occur within the cell.

## Background

In common with other signalling proteins, PKC has been suggested to associate with caveolin-1 [1,2], a key component of caveoli and membrane rafts, that are proposed to exist as signalling platforms in the plasma membrane and elsewhere in the cell. However, where in the cell the PKC-caveolin interaction might occur and under what conditions remains unclear.

Caveolae are vesicular organelles are that are involved in a wide range of cellular functions, serving as platforms or rafts, wherein reside a wide variety of signalling molecules [3]. The caveolin proteins (caveolin-1, -2, and -3) act as the structural components of caveolae. They also function as scaffolding proteins and as such recruit numerous signalling molecules to caveolae where their activity is regulated. PKC is a signalling molecule of major importance in cells, which in the form of twelve isoforms, regulates numerous signalling cascades by virtue of its ability to phosphorylate target proteins that include receptors, G-proteins, ion channels as well as other kinases [4-6]. This leads to control of numerous cellular processes, such as secretion, proliferation, differentiation, apoptosis, permeability, migration, hypertrophy etc [4,5,7-11].

While it has been shown that isolated caveoli interact with purified PKCα [12], PKCγ [13] and PKCε [14] using immunoprecipitation, where in the cell this occurs is not known. Caveolin contains a sequence that is a consensus site for phosphorylation by PKC [15], while down-regulation of plasma membrane-translocated PKCα involvesinternalization of the active enzyme that involves ubiquitination, through a caveolae-dependent mechanism, followed by multisite dephosphorylation and down-regulation in a perinuclear compartment in a time dependent manner (~30 min after stimulated translocation to the plasma membrane) [16]. It has also been shown that endocytic trafficking via caveolae may be a PKCα-dependent process [17]. These observations lead to the question of whether PKCα interacts directly with caveolin, and where in the cell this occurs, a question we examined in this present study.

The classic biochemical or immunoprecipitation approaches for determining the location of signalling molecules in cells, while commonly used, is severely limited for several reasons. The main drawback is that it involves destruction of the cell, resulting in loss of spatial information. Staining the cells with fluorescent antibodies provides a useful advance enabling apparent "co-localisation" to be obtained when two different fluorophores are used. However, the two fluorophores may still be a considerable distance apart without any protein-protein interaction between the pair occurring. The method also suffers from problems of photobleaching and when the probes are in abundance in the cell false co-localisation data can result.

Recently, with the advent of green fluorescent protein (GFP) technology, considerable new information has become available using imaging approaches. This allows the protein of interest to be tagged by expression in the cell, allowing it to be functionally located within the cell without recourse to antibodies. Numerous papers have shown signalling molecules in cells can be tracked as they move between subcellular locations using expression of GFP-tagged proteins and fluorescence microscopy. PKC has been studied in a wide range of situations using GFP-tags (e.g. [18-20]. Lifetime imaging has been used as a localisation tool for GFP-tagged proteins [21-23] and using this approach both PKCα activation levels, along with localisation, has been detected through the binding of fluorescently tagged phosphorylation site-specific antibodies using fluorescence energy transfer (FRET), measured through a donor fluorophore on the PKC [24]. In most cases the localisation was either followed in real-time or after fixation, following initiation of intracellular signalling. There are now a number of different fluorescent proteins available in the "GFP" family, for example cyan fluorescent protein (CFP), yellow fluorescent protein (YFP), red fluorescent protein (DsRed) etc.

A considerable advance over the co-localisation approach is to use steady state FRET between suitably tagged proteins. A further improvement over steady state FRET is achieved by monitoring the lifetime of the donor, which is independent of changes in concentration, photobleaching and various limitations over intensity-based detection. Donor lifetime quenching is evidence for a direct physical interaction and in addition does not require corrections due to spectral over-lap that are required in steady state FRET. The use of this approach FRET-FLIM for localisation using different GFP-type constructs has been described recently in the literature [25-27]. This approach in combination with 2-photon (2P) excitation provides a powerful imaging technique. It does not require complex corrections that intensity-based FRET entails, allows excitation within a narrow focussed plane without contributions outside this region, as would be the case in one-photon excitation, as well as other advantages, such as the ability to image deeper in tissue etc.

In this work the interaction of PKCα with caveolin, was investigated using two-photon-fluorescence lifetime imaging (2P-FLIM). This was determined by investigating the FRET between GFP-tagged PKCα and DsRed-caveolin (DsRed-cav). Using the quenched lifetime of the GFP-tag, areas showing co-localisation in CHO cells was identified after activation of the PKC had occurred. When the GFP-PKC is induced to translocate to membranes, excitation of the GFP at 850 nm, for 2P excitation, should lead to FRET to the DsRed construct if the two are co-localised. This would be seen as a reduced GFP lifetime, in contrast to areas in which the GFP-PKC resides but not with caveolin. This information was determined using a FLIM time-correlated single photon counting set up (TCSPC) with the frequency doubled output of a Tsunami pulsed laser (110 fs), coupled to an inverted microscope. Based on the lifetime quenching data the results were consistent with three populations of activated GFP-PKC. One within the cytoplasmic area of the cells, which was probably undergoing a direct interaction with caveolin, likely in endosomes, a second population in the cytoplasm and in the nucleus that was not interacting with caveolin and a third at the plasma membrane possibly indirectly interacting with caveolin.

## Results

**Figure 1:**
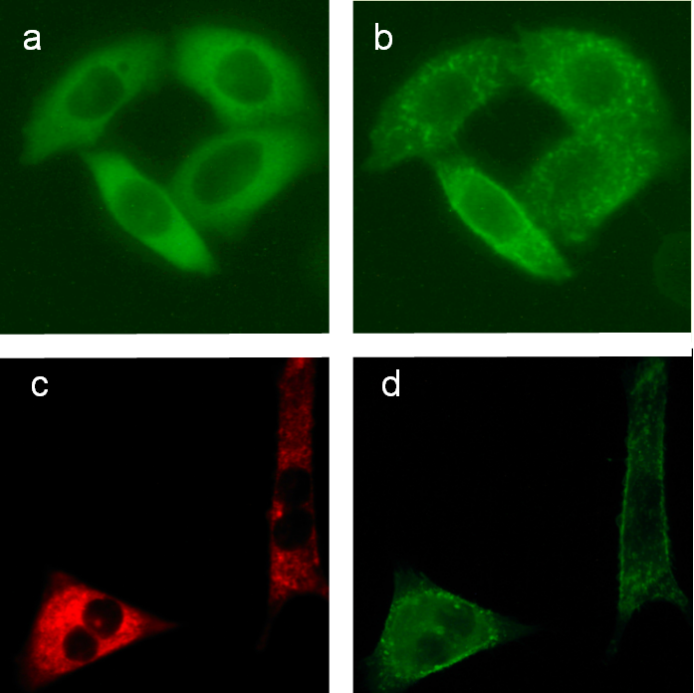
**Fluorescence intensity images of GFP-PKC and DsRed-cav expressed in CHO cells. **GFP-PKC and/or DsRed-cav were transiently expressed in CHO cells on glass coverslips by culturing for 48 hr. The cells were then treated with Ca^2+^-ionophore (1 nM) or TPA (100 nM) for 3 min before the cells were fixed on microscope slides as described under Methods. Images were acquired using a Olympus IX-70 inverted epifluorescence microscope with a 60× objective and GFP/DsRed filter sets and a Olympus C3030 camera. (a) a representative image before and (b) after Ca^2+^-induced translocation by ionophore of GFP-PKC to the peripheral membrane and to discrete regions in the perinuclear region. (c) image of DsRed-cav and (d) GFP-PKC, after treatment with 100 nM TPA.

The epifluorescence images in Figure 1 show GFP-PKC and DsRed-cav localisation as green and red colours respectively. The images were taken before and after exposure to the phorbol ester TPA for 3 min. It can be seen that PKC accumulated at the cell periphery and in organelles in the perinuclear region.

**Figure 2:**
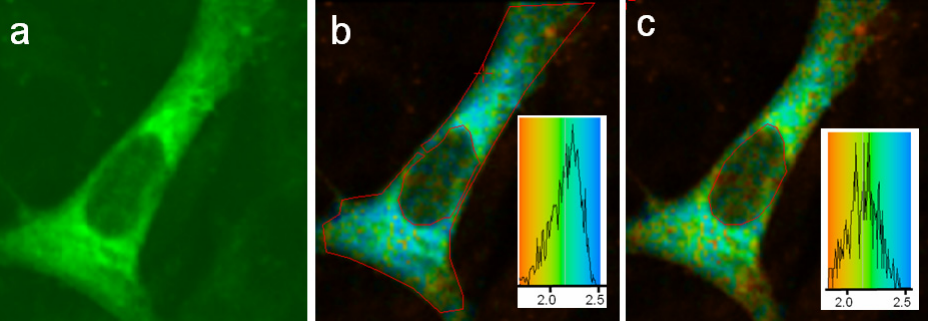
**Fluorescence lifetime imaging of GFP-PKC expressed in CHO cells. **GFP-PKC was transiently expressed in CHO cells as described in the legend in Figure 1 and detailed under Methods. Images were acquired using a Nikon 2000 inverted epifluorescence microscope with a 60× objective and GFP/DsRed filter sets and an ICAM camera. The 2P-FLIM images were collected using the TCSPC fast scanning imaging mode as described under Methods. (a) Conventional epifluorescence image of GFP-PKC distribution in a resting CHO cell, (b) lifetime image of the same cell with the analysis area enclosed by the red line (cytosol) shown (with colour coding) in the *inset *and giving an average lifetime of ~2.2 ns and (c) with the analysis area enclosed by the red line (nucleus) shown in the *inset *giving an average lifetime of ~2.0 ns. Cells shown are representative images from replicate experiments.

A lifetime image of a CHO cell transiently transfected with GFP-PKCα for 48 hr is shown in Figure 2, with the corresponding epifluorescence image. It can be seen that PKCα is widely and abundantly distributed in the cell cytoplasm and nucleus. The data collection time for this and other lifetime images was optimally 2–3 minutes. The lifetime data for the various conditions examined is also summarised in Table 1.

**Table 1:** Summary of average lifetimes for GFP emission under various conditions

**Transfection**	**Fig**	**Treatment**	**Area analysed**	Avg Lifetime (ns)
GFP-PKC	2	None	cytoplasm	2.2
		None	nucleus	2.2
	3	TPA	cytoplasm	2.2
		TPA	nucleus	2.3
GFP-PKC/DsRed-cav	4	None	cytoplasm	2.4
		None	cytoplasm	2.5
	5	Ca^2+^	cytoplasm	1.6*
		Ca^2+^	nucleus	2.0
	6	TPA	cytoplasm excluding peripheral	1.5*
		TPA	nucleus	2.0
		TPA	peripheral	1.8*
	7	Bradykinin	cytoplasm	1.6*

*reduced lifetime indicating PKC-GFP-DsRed-cav interaction

The lifetime of the GFP averaged for the cell area was around 2.2 ns (Figure 2b). The software analysis program optionally allows for whole or part of the field to be subjected to a lifetime analysis. By this means contributions from the background can be effectively excluded, as well as different areas with the cell compared. It is also possible to analyse the lifetimes on a pixel by pixel basis in discrete areas. Both approaches were used in this study. If the analysis was not restricted to the cell area and the whole field is included the background contributions became significant and a background component with a lifetime of ~1.4 ns became apparent. A single pixel analysis, within the cell area, yielded 2.0 ns for a single exponential with a reduced χ^2 ^of 1.22, a two exponential analysis did not give an improved fit. Based on this, and observations from the literature, we analysed the lifetime of GFP as a single exponential. Analysis of the GFP-PKC located within the nucleus showed the same lifetime as that in the cytoplasm (Figure 2c).

**Figure 3:**
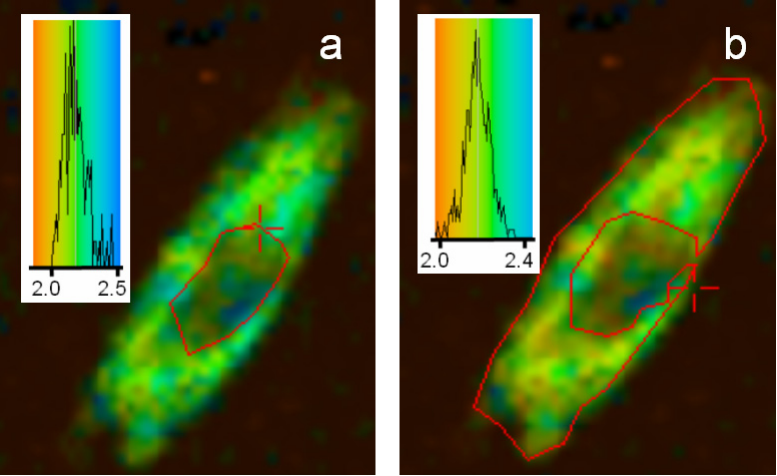
**Fluorescence lifetime imaging of GFP-PKC expressed in CHO cells: effect of TPA. **2P-FLIM images were collected as described in the legend to Figure 2 except cells were treated with TPA (100 nM) for 3 min. Treatment with the phorbol ester did not affect the fluorescence lifetime of GFP attached to PKC. (a) Fluorescence lifetime image with the analysis area enclosed by the red line (nucleus) shown in inset giving an average lifetime of ~2.1 to 2.2 ns. (b) Fluorescence lifetime image with the analysis area enclosed by the red line (cytosol) shown in inset giving an average lifetime of ~2.2 ns. Insets: lifetime distributions with colour coding.

We next examined the effect of the phorbol ester TPA on the GFP-PKC lifetime. If a FRET analysis is to be usefully undertaken it is first essential that while the PKC may redistribute in the cell, as a result of the TPA treatment, it should not result any changes to the lifetime. This was in fact the case as shown in Figure 3 where the lifetime for GFP-PKC across the cell area remained unchanged in the ~2.2 ns region.

**Figure 4:**
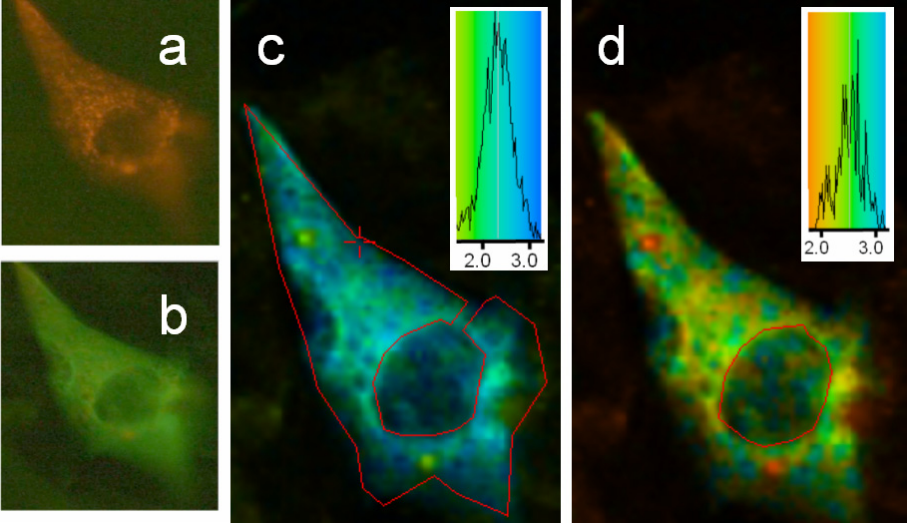
**Fluorescence lifetime imaging of GFP-PKC co-expressed with DsRed-cav in CHO cells. **2P-FLIM images were collected as described in the legend to Figure 2. Co-expression of the GFP-PKC with DsRed-cav does not affect the lifetime of the GFP showing that in the unstimulated state PKC is not associated with caveolin. Epifluorescence images for excitation of DsRed (a) and GFP along with DsRed (b) showing that the PKC and caveolin co-distributed in the cytosol. Fluorescence lifetime images with the analysis area enclosed by the red line, (cytosol) (c) or nucleus both essentially showing a lifetime as for Figs 2–3 centred around ~2.2 ns. Cells shown are representative images from replicate experiments.

The images in Figure 4 are from cells with GFP-PKC co-expressed with DsRed-cav. The epifluorescence images show that both PKC and caveolin were widely distributed in most cell areas, except that caveolin was not found in the nucleus and was concentrated in the perinuclear region. The lifetime images of GFP-PKC were acquired under conditions that would produce no contributions from DsRed fluorescence. This was achieved since the 2-P excitation was nominally at 425 nm, where there is virtually no excitation of the DsRed, also a 500 nm band-pass filter was used to exclude DsRed emission. Therefore only the GFP emission was collected. If the DsRed were to be close enough to PKC then a shortened lifetime would result due to FRET occurring. Again the average lifetime of the GFP-PKC was >2 ns, showing that under conditions when the cell is not stimulated (as defined here by Ca^2+^-mobilisation or phorbol ester activation of PKC), then PKC and caveolin do not co-associate in the cell.

**Figure 5:**
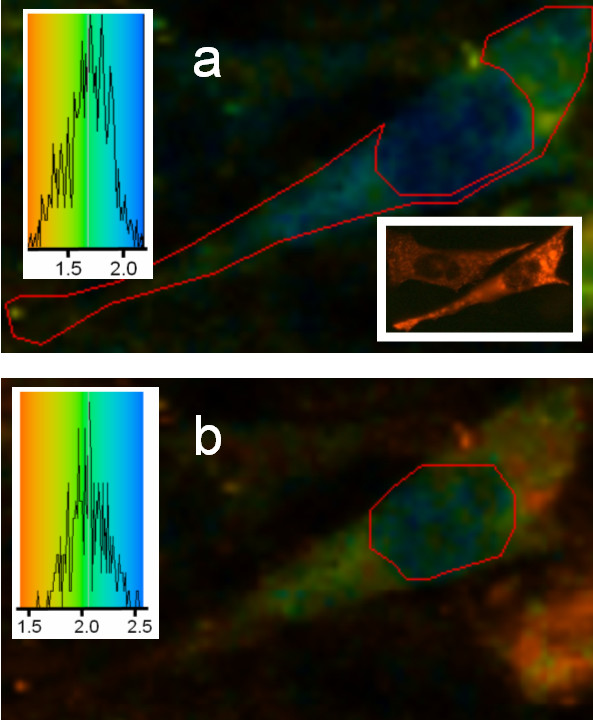
**Lifetime imaging of GFP-PKC co-expressed with DsRed-cav in CHO cells: effect of Ca2+-ionophore. **2P-FLIM images were collected as described in the legend to Figure 2. Cells were treated with ionophore for 3 min before mounting and fixation as described in Methods. The epifluorescence image in the inset shows the DsRed-cav distribution (cytoplasmic) which was not affected by the Ca^2+ ^ionophore. When the cytoplasmic area was analysed, (a) as shown by the area within the red line, both orange and green/blue areas are seen indicating the presence of both GFP-PKC and quenched GFP-PKC – note that only GFP lifetime can be observed in the lifetime images. This indicates that DsRed-cav was sufficiently close to the PKC-GFP to induce a quenching of the GFP by the DsRed, i.e. the PKC is translocating to caveolin containing areas. By contrast, in the nucleus (b) only GFP-PKC was expressed and the lifetime was unquenched (~2.2 ns). This is the same as the lifetime for GFP-PKC when only the latter is expressed (see Figure 2). Cells shown are representative images from replicate experiments.

When Ca^2+ ^ionophore is added to cells Ca^2+ ^gains entry and this induces immediate PKC translocation to various locations within the cells, including the perinuclear regions and peripheral membranes, as has been extensively shown in the literature (e.g. see ref [28,16,29]). The localisation of caveolin did not appear to change upon addition of Ca^2+ ^ionophore to the cells (results not shown). For lifetime images it is important to note that what we are observing is the lifetime of the GFP, not its intensity. The lifetime images shown in Figure 5 show that the GFP-PKC fluorescence lifetime in the cytoplasm is reduced to ~1.6 ns. This is due to the quenching by DsRed, since the lifetime is unaffected in the absence of DsRed-cav. By contrast, the GFP-PKC lifetime in the nucleus was unaffected by the Ca^2+ ^treatment, since there was little or no caveolin within the nucleus (Figure 5).

**Figure 6:**
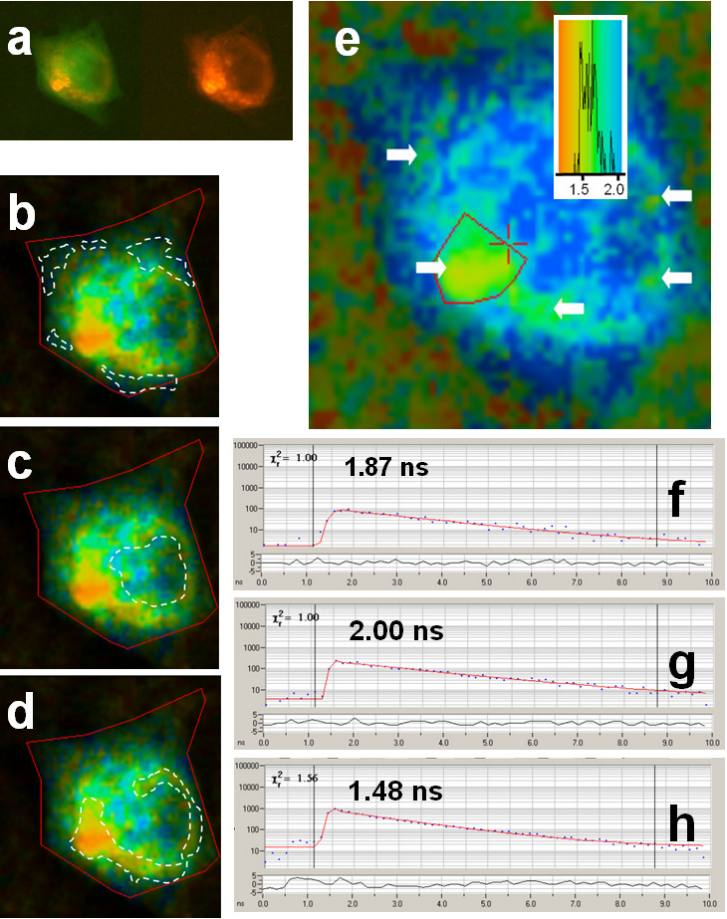
**Lifetime imaging of GFP-PKC co-expressed with DsRed-cav in CHO cells: effect of phorbol ester. **Epifluorescence and 2P-FLIM images were collected as described in the legend to Figure 2. Cells were treated with TPA (100 nM) for 3 min before mounting and fixation as described in Methods. (a) left: GFP-PKC and DsRed-cav co-distribution revealed in a green and red epifluorescence image (red and green filters) and right: DsRed-cav visualised using a red filter, the caveolin was mainly restricted to the perinuclear region with PKC more widely distributed. Three distinct GFP lifetimes are discernable in the lifetime image and were separately analysed. For the lifetime images shown in (b, c and d), representative single point analyses within the regions enclosed by dashed white lines are analysed in (f, g and h) respectively, as follows: (b and f) peripheral regions (τ avg: 1.8 ns; single point 1.87 ns [χ^2 ^1.00]); (c and g) the nuclear region (τ avg: 2.0 ns; single point 2.00 [χ^2 ^1.00]); (d and h) the cytoplasm (τ avg: 1.5 ns; single point 1.48 ns [χ^2 ^1.56]). (e) example of derivation of average lifetime for one of the three areas, shown for the cytoplasm, with the analysis for the area enclosed in red (lifetime colour coding shown in the inset) and other similar areas in the cytoplasm indicated by white arrows. Cells shown are representative images from replicate experiments.

The treatment of the cells with the phorbol ester TPA not only induces translocation of PKC to different cell compartments but also produces catalytically active PKC. In general, PKC moves to the outer membrane and to perinuclear regions, and associates with various signalling and cytostructural components in the cell. The lifetime image data revealed that, similar to the effect of increasing intracellular Ca^2+^, phorbol ester induced PKC to associate with caveolin, the GFP lifetime being reduced accordingly, as shown in Figure 6.

Three discrete areas could be ascertained from visual inspection of the FLIM images. Unfortunately when such areas are relatively small or scattered it is not possible to use the masking option of the software as used here to restrict analysis to the nucleus or cytoplasm since the data becomes noisy. Therefore a single pixel analysis within such areas has to be performed. These were found at the cell peripheral regions, the nuclear region and the perinuclear region (Figure 6b–d), These areas are outlined with dotted white lines. Figure 6f–h shows representative *single pixel *analyses for each of the three regions. The lifetimes (single exponential) were 2.0 ns (nuclear), 1.87 ns (peripheral) and 1.48 ns (perinuclear). This indicates that FRET, due to co-localisation of the caveolin and PKC, is predominantly occurring in the perinuclear region (see also analysis of a representative perinuclear *region *(depicted with white arrows in Figure 6e), and to some extent in the peripheral regions of the cell, with little or no co-localisation within the nuclear region, due to a lack of significant caveolin in that region.

**Figure 7:**
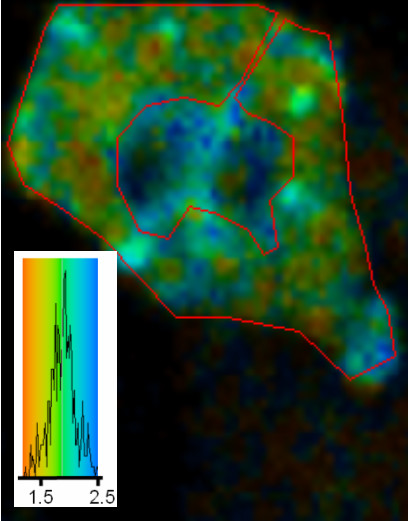
**Lifetime imaging of GFP-PKC co-expressed with DsRed-cav in CHO cells: effect of bradykinin. **2P-FLIM images were collected as described in the legend to Figure 2. Cells were treated with 5 nM bradykinin for 3 min before mounting and fixation as described in Methods. The area in the cytoplasm outlined in red was analysed as shown in the colour coded inset (τ avg: 1.6 ns), showing PKC interaction with caveolin.

The effect of the hormone bradykinin was also examined, a hormone that interacts with a G-protein coupled receptor and initiates intracellular Ca^2+^-release and also the production of DAG. Again the lifetime images (Figure 7) showed a reduced lifetime for GFP indicative of FRET to the DsRed and a co-localisation.

## Discussion

Caveolin is the main structural component of caveoli and in addition performs an important role as a scaffold protein interacting with many key cellular signalling components [1,2]. In addition caveoli constitute a special class of membrane rafts, discrete areas in the cell membrane, within which related signalling molecules co-localise to optimise signalling events [30]. In other studies we have addressed the question whether PKC interacts with cell membrane rafts and have found that as a result of phorbol ester treatment the different PKC isoforms distribute into these regions, as defined by recovery in a detergent resistant fraction. (Slater, S. J. *et al*., unpublished observations) In the present study, we show for the first time, using a FRET-FLIM approach, that PKCα and caveolin co-localise upon increased intracellular Ca^2+ ^or phorbol ester-induced interaction with PKC, however, in the 'resting' state the two molecules do not interact.

The translocation of PKC to various regions in the cell from a basically cytosolic location upon activation is a basic feature of signalling involving this molecule, and is important to many cell processes. Earlier studies considered that the main effects acted through IP_3_-induced release of intracellular Ca^2+^, induced by hormone stimulated G-protein coupled receptor-linked G-protein-induced stimulated release of IP_3 _and diacylglycerol (DAG) induced by the action of phospholipase C on phosphatidylinositol 4,5-bisphosphate. The PKC would then induced by the Ca^2+ ^to move to the membrane, following which it would encounter with DAG which induces PKC to unfold and expose its substrate binding site which is followed by its phosphorylation. When PKCα is exposed to Ca^2+ ^released from internal Ca^2+ ^stores it transiently translocates to the outer membrane but returns to the cytoplasm [20,31], unless the activator levels are sustained, as occurs under the conditions used in the present work. It is now clear that PKC interacts with other cellular structures and organelles, including actin and RhoA for example [32,33]. Thus it is possible for a single isoform to be simultaneously involved in several distinct processes in the cell in different locations. Therefore to understand, and eventually bring under some form of control in therapeutic situations, new methodologies are required to unravel such complications. The combined approaches of fluorescence imaging and GFP-technology is now revolutionising our understanding of complex signalling events in cells such as presented by elements such as PKC. Conventional imaging techniques have been widely used in studies of signalling events in cells. Due to the equipment being widely available and inexpensive conventional epifluorescence imaging is widely utilised. However, due to problems such as photobleaching, spectral overlap, and lack of information on concentrations etc the information that can be gained is limited and often over-interpreted. A major question in cell biology is currently whether one particular protein interacts with another. In the present study using a 2P-FLIM-FRET approach we were able to unequivocally demonstrate that PKC moves to caveolin containing structures as a result of interaction with Ca^2+ ^or phorbol ester.

The principle caveolin containing structures in the cell are caveoli, which transport to and from the plasma membrane and endosomes [16]. In the present work we showed that PKC translocates in a Ca^2+^-dependent manner. To examine this we used a 3 min time point. From other studies on CHO cells (Kelly, M. *et al*., in preparation) we have found that PKC translocation takes place over a 0–3 min time frame after which the cell morphology begins to change. Therefore for the purposes of this study, which was to prove the technology and to find out if PKC and caveolin interact, the 3 min time frame was optimal. It is important to note that PKC can translocate to a region as a result of Ca^2+ ^interaction but this does not mean that it is catalytically active. It then has to encounter DAG or other signalling elements in order to gain phosphorylation ability. The nature of this element and whether DAG is produced in these regions of the cell has yet to be determined. In addition we do not yet know the role of PKC in endosomes or what other signalling molecules may be involved. It is possible that in the normal of the cell PKC is directed to endosomes as a part of a degradation pathway but this also remains to be determined.

In order to perform FLIM-FRET measurements it is important to determine that there are no spurious effects on the GFP donor lifetime properties. Here we showed that the translocation of PKC to its various locations in the cell had no effect on the GFP excited state lifetime in the absence of acceptor. It is also important to note that if there was a *distribution *of close GFP-DsRed distances, then a range of GFP decays for varying quenching would have occurred and a multi-exponential fit to the data would give an improved fit to the data. On the contrary, a single exponential fit to the data was optimal under FRET conditions indicating that the GFP and DsRed were in close contact. The next requirement is that since, compared to bulk fluorescence measurements, the signal is necessarily relatively weak, contributions from background and other factors must be minimized. In this study we found that the background interfered if the entire cell field was analysed. However if the cell region was isolated using the masking option in the software the background contribution could be eliminated. This can also be achieved by increasing the pixel resolution. Further, specific regions within the cell must be discerned for the imaging approach to be useful. Here we were able to isolate three discrete regions in the cells. These were PKC-GFP in the plasma membrane/peripheral regions, the perinuclear region (endosomes) and the nuclear region (see Table 1). The lack of caveolin in the nucleus allowed an internal control for each cell analysed and no quenching of the GFP lifetime was found in that region. In the endosome region significant quenching could be seen with the lifetime dropping from ~2.2 to ~1.5 ns. This is indicative of a direct interaction between the GFP and DsRed, attached to the PKC and Caveolin proteins. The peripheral region of the cell also showed a reduced GFP lifetime but by a lesser degree than in the perinuclear region. This could be due to the interaction being indirect but still close enough to allow FRET. Since FRET falls off in intensity according to the inverse forth law it will still have to be close but perhaps with a small intermediate protein. These possibilities remain to be further explored. Finally in this study we chose a point in time rather than following live cells since the PKC locality stabilised over a 2–3 min time course and could be conveniently analysed when the cells were fixed and mounted on slides. There is no indication, however, that the signal would be insufficient to follow time-dependent changes as collection times as low as 15 sec. could be used still allowing a signal sufficient for lifetime analysis.

## Conclusion

In conclusion we have shown that PKC translocates to caveolin as a result of Ca^2+ ^or phorbol ester interactions. The 2P-FLIM-FRET approach combined with GFP-technology offers a method for unambiguously determining the location of specific protein-protein interactions within the cell. Here we show that PKC translocates to and interacts with caveolin.

## Methods

Chinese hamster ovary (CHO) cells were obtained from the ATCC (Manassas, VA) or were generously provided by Dr. Emma Leatherbarrow (MRC, Harwell, UK). Cells were cultured on glass cover slips in F-12 HAM's media (Sigma-Aldrich) supplemented with 10% Foetal calf serum (ICN Biomedicals or Gibco), 1% L-glutamine and 1% penicillin/streptomycin (Sigma Aldrich or Gibco) and cultured until reaching confluence.

For transfection 1–2 μg of pPKCα-EGFP vector DNA (Clontech) and/or DsRed1-cav-1, (kindly provided by Dr. R. Pagano [34], Mayo Clinic and Foundation) was added to cells in Lipofectin (Invitrogen) according to the manufacturer's protocol. Cells were incubated for a further 48 h and were then washed with phosphate buffered saline (PBS). Cells were then treated with 4β-12-O-Tetradecanoylphorbol-13-acetate (TPA), calcium ionophore or A23187, bradykinin (all from Sigma-Aldrich), as indicated, or were untreated (controls). The cells were then fixed in 3.7% formaldehyde solution in PBS for 20 minutes at room temperature washed again with PBS before mounting on slides with Crystal Mount before FLIM analysis.

Epifluorescence images were collected using a Nikon TE2000 U or Olympus XI microscope and a GFP or DsRed filter cube set and a 60× objective. Fluorescence lifetime images were obtained using a 2P-microscopy apparatus, using the Nikon microscope, constructed in the Central Laser Facility of the Rutherford Appleton laboratory, which has a Bio-Rad MRC600 confocal scanning system or external x, y galvanometers, [35,36]. Laser light at a wavelength of 850 nm was obtained from a Titanium Sapphire, 82 MHz, mode-locked laser (Spectra-Physics), with a pulse width of 120 fs. The light was focused to a diffraction-limited spot through an air (x40, n.a. 0.9) and specimens illuminated at the microscope stage by passing the beam through the MRC600 scan head or through a dichroic at the epifluorescence port.

Fluorescence emission was passed through a BG39 filter (Comar) to remove the laser line. The scan was operated in the normal mode, and line, frame and pixel clock signals were generated and synchronized with an external fast micro channel plate – photomultiplier tube, which was used as the detector (Becker & Hickl, GmbH Germany). These were linked via a Time-Correlated-Single Photon Counting PC module SPC700 (Becker & Hickl). With this system we were able to distinguish distinct lifetime regions within a single cell provided the lifetimes differed by greater than 0.2 ns. The set up was used to excite GFP fluorescence, the lifetime of which was measured as a function of the quenching by DsRed when the GFP-PKC and DsRed-cav were close enough for fluorophore tags to participate in FRET.

Images (6 bit, 256 × 256 pixels) were exported from Becker & Hickl software as bitmaps and converted into TIFF files. Image analysis was performed using either Irfanviewer or Image Pro 5.1 software (steady state), FLIM analyses were performed using SPC Image 2.0 software (Becker & Hickl).

## Authors' contributions

CDS designed and performed all experiments and analyses and drafted the manuscript. SWB supervised the data collection instrumentation helped draft the manuscript. SJS helped to design the experiments and supervised pilot fluorescence studies. AWP and SWB designed and constructed the apparatus used for fluorescence lifetime measurements. AWP helped draft the manuscript. All authors have read and approved the manuscript.
